# Neuro-musculoskeletal simulation of instrumented contracture and spasticity assessment in children with cerebral palsy

**DOI:** 10.1186/s12984-016-0170-5

**Published:** 2016-07-16

**Authors:** Marjolein Margaretha van der Krogt, Lynn Bar-On, Thalia Kindt, Kaat Desloovere, Jaap Harlaar

**Affiliations:** Department of Rehabilitation Medicine, VU University Medical Center, MOVE Research Institute Amsterdam, PO Box 7057, 1007 MB Amsterdam, The Netherlands; Department of Rehabilitation Sciences, KU Leuven, Tervuursevest 101, B-3001 Leuven, Heverlee Belgium; Clinical Motion Analysis Laboratory, University Hospital Leuven, Weligerveld 1, 3212 Pellenberg, Belgium

**Keywords:** Muscle spasticity, Muscle stiffness, Neuro-musculoskeletal modeling, Cerebral palsy, Biomechanics, Electromyography, Rehabilitation

## Abstract

**Background:**

Increased resistance in muscles and joints is an important phenomenon in patients with cerebral palsy (CP), and is caused by a combination of neural (e.g. spasticity) and non-neural (e.g. contracture) components. The aim of this study was to simulate instrumented, clinical assessment of the hamstring muscles in CP using a conceptual model of contracture and spasticity, and to determine to what extent contracture can be explained by altered passive muscle stiffness, and spasticity by (purely) velocity-dependent stretch reflex.

**Methods:**

Instrumented hamstrings spasticity assessment was performed on 11 children with CP and 9 typically developing children. In this test, the knee was passively stretched at slow and fast speed, and knee angle, applied forces and EMG were measured. A dedicated OpenSim model was created with motion and muscles around the knee only. Contracture was modeled by optimizing the passive muscle stiffness parameters of vasti and hamstrings, based on slow stretch data. Spasticity was modeled using a velocity-dependent feedback controller, with threshold values derived from experimental data and gain values optimized for individual subjects. Forward dynamic simulations were performed to predict muscle behavior during slow and fast passive stretches.

**Results:**

Both slow and fast stretch data could be successfully simulated by including subject-specific levels of contracture and, for CP fast stretches, spasticity. The RMS errors of predicted knee motion in CP were 1.1 ± 0.9° for slow and 5.9 ± 2.1° for fast stretches. CP hamstrings were found to be stiffer compared with TD, and both hamstrings and vasti were more compliant than the original generic model, except for the CP hamstrings. The purely velocity-dependent spasticity model could predict response during fast passive stretch in terms of predicted knee angle, muscle activity, and fiber length and velocity. Only sustained muscle activity, independent of velocity, was not predicted by our model.

**Conclusion:**

The presented individually tunable, conceptual model for contracture and spasticity could explain most of the hamstring muscle behavior during slow and fast passive stretch. Future research should attempt to apply the model to study the effects of spasticity and contracture during dynamic tasks such as gait.

**Electronic supplementary material:**

The online version of this article (doi:10.1186/s12984-016-0170-5) contains supplementary material, which is available to authorized users.

## Background

Cerebral palsy (CP) is the most common neurological disorder in children and is attributed to non-progressive disturbances occurring in the developing fetal or infant brain [1]. It is characterized primarily by neural deficits (caused by the brain anomalies) and secondary by muscular and bone deformities [2, 3]. These deficits adversely affect normal development of functional activities, such as gait. Muscle hyper-resistance, i.e. increased tension in a passively stretched muscle, caused by neural impairments (spasticity and increased background activation) as well as by passive tissue stiffness, is the most prevalent problem. Spasticity is commonly defined as a velocity-dependent increase in tonic stretch reflexes due to hyper‐excitability [4], while stiffness is a mechanical resistance of the myotendinous tissue as it is passively lengthened [5].

The exact pathophysiology of hyper-resistance is complex and only partially understood. Specifically, there is lack of consensus on the mechanisms of, and interaction between, the neural and non-neural components of hyper-resistance. For example, it is generally thought that muscle stiffness occurs secondary to spasticity, with prolonged activation causing short and stiff fibers [6]. Compared with muscles in typically developing (TD) children, muscles in CP are smaller in cross sectional area, volume and muscle belly length, while Achilles tendons may be longer [7, 8]. However, studies on passive fascicle length and pennation angles have been inconclusive [9, 10], with some results indicating normal fascicle lengths [11] and long sarcomeres in spastic muscles [12]. Recent studies also suggest that muscle stiffness in CP already appears at an early age [13] and that maladaptation to growth, rather than spasticity, plays a crucial role in developing contractures [14].

The medial hamstrings are frequently affected by hyper-resistance in CP. In comparison to TD muscles, spastic hamstrings have been found to have lower activation thresholds during passive stretch [15] as well as altered muscle properties, including increased muscle stiffness due to larger amounts of extracellular matrix [16]. Recently, instrumented clinical tests to assess hyper-resistance in the hamstrings have been developed, with which both the biomechanical and electrophysiological responses are recorded during a passive stretch [17, 18]. Such tests yield a vast array of information about joint resistance, joint angles, angular velocity, and muscle electromyography (EMG). However, (instrumented) clinical assessment provides limited information at the underlying muscle level. This makes it difficult to distinguish between the neural and non-neural components of hyper-resistance, based on experimental data alone.

(Neuro-)musculoskeletal modeling is a powerful tool to gain insight into the underlying pathology at a more detailed level [19]. Thus far, most modeling studies of spasticity during passive stretch have focused on the ankle joint and have applied either simplified [20, 21], or highly complex [22–25] muscle models. Other modeling approaches have attempted to directly [26, 27] or indirectly [28] assess the effect of stretch reflexes on gait. However, the complexity of pathological muscle behavior during voluntary activation makes it very challenging to validate such models with measured data. A detailed analysis of spastic hamstring behavior during passive instrumented clinical assessment using neuro-musculoskeletal modeling could yield valuable information on tissue as well as reflective muscle behavior, but is so far lacking in literature.

Therefore, the aim of this paper was to simulate instrumented, clinical spasticity assessment of the hamstring muscles in CP using a conceptual model of contracture and spasticity. Specifically, we investigated whether we could explain 1) increased resistance during slow passive stretch (i.e. contracture) by altered passive muscle stiffness; and 2) additional increased resistance during fast passive stretch (i.e. spasticity) by a purely velocity-dependent stretch reflex.

## Methods

Eleven children with spastic CP (6 male, age 11.5 ± 3.4y; weight 33.7 ± 12.8 kg) and 9 TD children (5 male; age 11.0 ± 3.2 y; 34.8 ± 13.5 kg) were included in the study. Of the CP subjects, 8 were bilaterally affected and 3 unilaterally; 8 were classified as Gross Motor Function Classification System (GMFCS) level I and 3 as II. All data were collected as part of a larger study [29]. Patients were selected from the available data set if the Modified Ashworth Scale score of the hamstring muscles was 1 or higher [30], i.e. a clinical sense of mild hyper resistance (1 subject had a score of 1; 6 of 1+, and 4 of 2; with an average Modified Tardieu Score of −78.5 ± 6.7°). Only data on left legs were included for practical reasons. To exclude patients with high background activation or who were not relaxed, measurements with a root mean square EMG (RMS-EMG) activity during slow passive stretch higher than 10 % of that measured during maximum voluntary contraction (about 2 % of the stretches) were excluded. Available data of 9 TD children were used for comparison [17]. All subjects older than 11 years and all parents signed an informed consent form. The data collection protocol was approved by the medical ethics committee of the KU Leuven University Hospital.

**Fig. 1:**
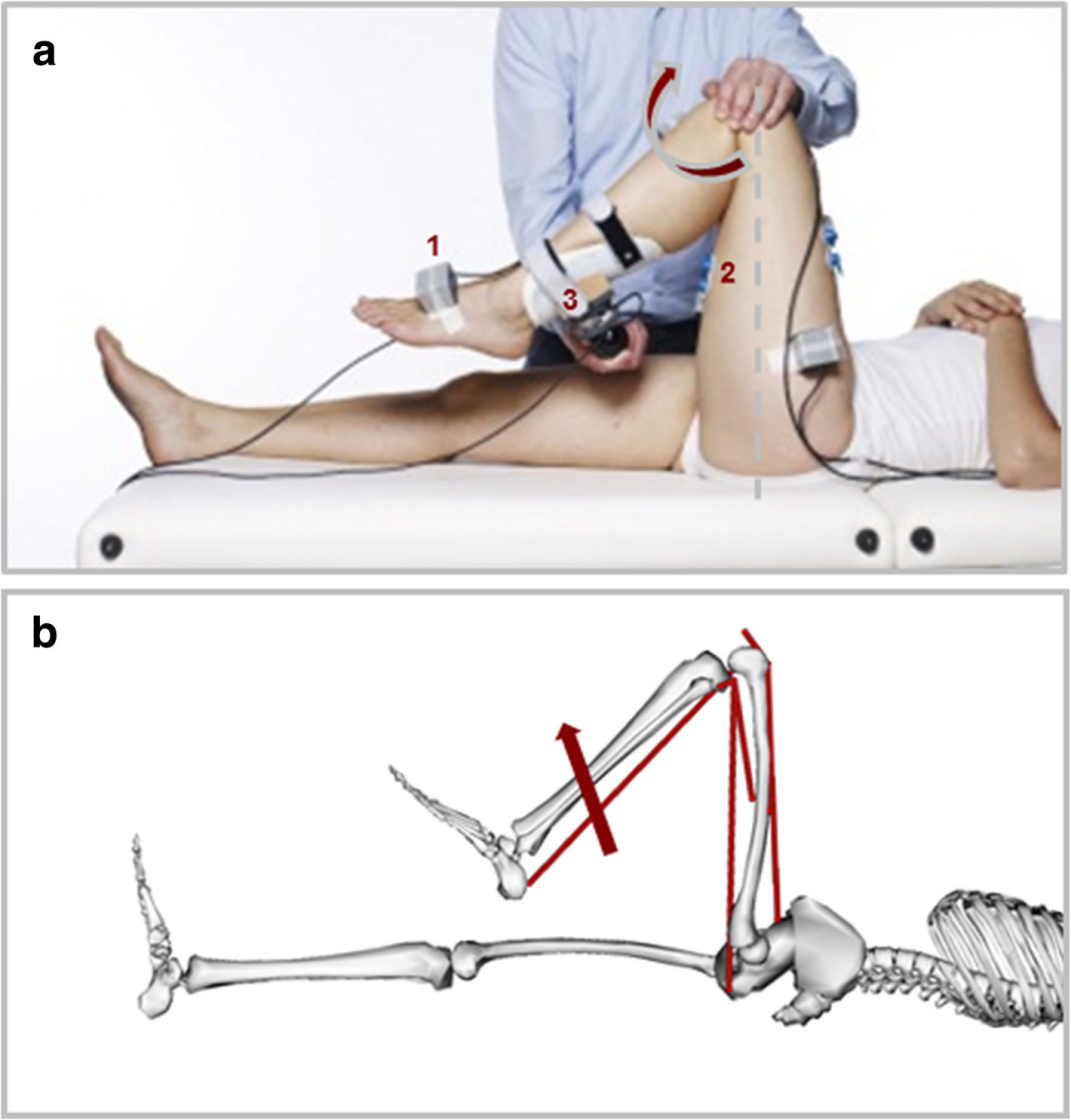
Measurement and model set-up. **a** Measurement set-up showing (1) inertial sensors, (2) EMG electrodes and (3) hand-held 6 - DOF force transducer; **b** OpenSim model used with 5 lumped muscles around the knee, and the applied force as measured experimentally

Instrumented spasticity assessment of the left leg hamstrings was performed on all subjects (Fig. 1a; [17]). The knee was stretched at a slow (>5 s for the entire range of motion) and a fast (<1 s) stretch velocity, while the subject was laying supine with the left hip in 90°, and the pelvis and thigh fixed by a second examiner. Knee angular displacement and velocity were recorded with inertial measurement units. The force applied to the shank by the examiner was recorded with a hand-held 6 degrees of freedom (DOF) force/torque sensor. The location of force application relative to the knee joint axis was measured manually parallel and perpendicular to the tibia. Surface EMG was collected from the medial hamstrings and rectus femoris (see [17] for measurement details).

A dedicated musculoskeletal model (Fig. 1b) was developed using OpenSim software [31]. The model was adapted from the generic gait model (GAIT2392) and included torso, pelvis, and left and right femur, tibia and foot (of which torso and right leg for visualization only). All joints were locked in 0°, except for the left hip, which was locked in 90° as imposed during all measurements, and the evaluated left knee, which was free to move until full extension. All muscles were removed except for those around the left knee, which were lumped to 5 major muscle groups: hamstrings (HAM, representing semitendinosus, semimembranosus and biceps femoris long head), vasti (VAS, representing vastus lateralis, medialis and intermedius), rectus femoris (RF), biceps femoris short head (BFS), and gastrocnemius (GAS, representing gastrocnemius lateralis and medialis). The path of the lumped muscles and the total muscle tendon length (optimal fiber length plus tendon slack length) were equal to that of one representative original muscle (biceps femoris long head for HAM, vastus intermedius for VAS, and gastrocnemius lateralis for GAS). Paths were selected based on other simplified models (e.g. Gait10dof18musc.osim) and previous literature [32]. To obtain myotendinous behavior representative of the whole muscle group, the fiber to tendon length ratio was averaged over all three muscles. The optimal muscle force was taken as the sum of the represented muscles’ forces. The ‘default activation’ was set to 0.01 for all muscles.

The model was scaled to individual subject sizes based on the subject’s height, leg length and tibia length, as measured during clinical examination. Muscle strength was scaled with the subject’s mass to the ^2^/_3_-power. Experimentally measured knee angles and applied forces were imported to OpenSim. The measured 6-DOF force was applied to the shank segment at the same location relative to the knee as measured experimentally. An inverse dynamic (ID) analysis was run in OpenSim to obtain the net knee moment, representing the sum of all (passive plus active) internal muscle moments around the knee.

As a model for contracture, passive muscle properties for HAM and VAS were optimized to match the slow-velocity net knee moment versus knee angle curve. The other three muscles were found to have negligible effect, as they were not in their end range of motion in any part of the assessment. The two parameters that determine the passive force-length curve in the Thelen muscle model [33] were adjusted, i.e. the parameters FmaxMuscleStrain (‘S’, the passive muscle strain at maximum isometric muscle force) and KshapePassive (‘K’, the exponential shape factor for the passive force-length relationship). An fminsearch optimization algorithm was used (Matlab 2010, The Mathworks), with the default values in OpenSim (S = 0.6; K = 4) as starting values, and no boundaries. To test the robustness of the optimization, additional different starting values were used, and this was found to have negligible effect. A muscle analysis was performed for each set of S and K, to obtain the total muscle knee moments generated by all five muscles. The sum of all muscle moments was compared with the net knee moment from the ID analysis. In the optimization, the RMS error between the two curves was minimized. The optimal S and K values for HAM and VAS, as well as the associated RMS error were obtained for each subject.

To test how well the model could predict measured motion, forward dynamic (FD) simulations were performed for all assessments. The measured initial position and applied forces were used as input, while predicted knee angle and muscle activity were output of the simulations. The passive muscle properties S and K were set to the optimal values for each individual for all FD simulations. First, the slow stretch data were simulated for both TD and CP, to verify that the model was capable of replicating data using FD simulations. Second, the fast stretch data were simulated to predict the effect of stretch reflexes in CP, and to obtain a reference in TD.

To model spasticity during the CP fast stretch simulations, a stretch reflex controller was developed in OpenSim. This controller imposed a velocity-dependent muscle excitation any time a fiber was stretched above a certain threshold velocity, according to:

$$ {E}_m\left(t+{t}_d\right)=\Big\{\kern1em \begin{array}{c}G\cdot {v}_m(t)\kern1em \Big|\kern1em {v}_m(t)>T\operatorname{}\kern1em \\ {}\kern3.7em 0\kern1.25em \Big|\kern1em {v}_m(t)\kern.5em \le \kern.5em T\operatorname{}\end{array}\kern1em \operatorname{} $$

with *E*_*m*_ the muscle excitation for muscle *m*, *t*_*d*_ a time delay factor representing the stretch reflex delay, *G* a gain factor representing the severity of the enhanced reflex, *v*_*m*_ the fiber velocity of muscle m, and *T* the threshold velocity. The controller was implemented as a plug-in in OpenSim and is freely available on https://simtk.org/home/spasticitymodel [34]. *t*_*d*_ was set to a fixed value of 30 ms, representing the shortest possible stretch reflex delay as found in the literature [35]. Based on the measured data, threshold values were determined as the stretch velocity 30 ms before onset of EMG of the muscle-tendon complex (thus assuming that muscle fiber velocity equals muscle-tendon velocity, which is reasonable considering the low forces). EMG onset was identified using the method of Staude and Wolf [36]. Gain values were set to 0, 1, 2 and 4 for each subject, which was found to cover the range of potential values. As no notable EMG activity was seen in any of the TD subjects’ fast stretches, the stretch reflex controller was only applied during CP fast stretch simulations.

To validate the FD results, predicted knee angles were compared with measured knee motion, and muscle excitation as evoked by the stretch reflex controller was compared with measured EMG. Both comparisons were done qualitatively by looking at the graphs, and for knee motion also quantitatively by calculating the RMS difference between the curves. A distinction was made between the stretch phase, in which the knee was moved quickly to its end range of motion, and the hold phase, in which the knee was held stationary at this end range of motion. In total 1 s of data was simulated for all FD simulations. The muscle fiber length and velocity during the CP fast stretch simulations were also analyzed, to gain further insight in the working mechanisms of the stretch reflex controller.

For statistical analysis, the optimal S and K parameters and the RMS error of the optimization were compared between TD and CP using a Mann–Whitney U test. This non-parametric test was chosen because of the small sample and non-normally distributed data. The RMS errors of the FD simulations for knee angle were also compared between TD and CP using a Mann–Whitney U test, and between different gain values in CP using a Kruskal-Wallis test. The level of significance was set to *p* < 0.05.

## Results

### Optimization of passive muscle properties

**Fig. 2:**
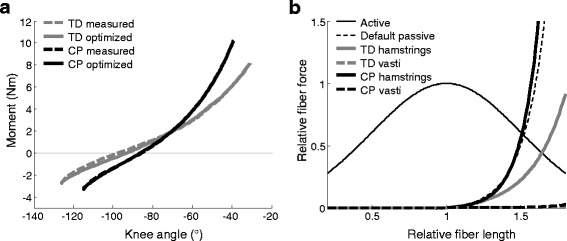
Knee moment-angle and muscle force-length curves. **a** Knee moment-angle curve for typically developing (TD, *grey*) and cerebral palsy (CP, *black*) children, averaged over all subjects. Inverse dynamic results of the measurement are shown (*dashed*), as well as the optimized results of the muscle analysis (*solid*). **b**. Active and passive muscle force-length curves, based on the optimized passive properties S and K as averaged over all TD (*thick grey*) and CP (*thick black*) subjects, for hamstrings (*solid*) and vasti (*dashed*). The TD vasti curve mostly underlies the CP vasti curve. Default active and passive curves (*thin lines*) are based on Thelen (2003) and typically assumed equal for all muscles, both only scaling in amplitude to maximum isometric muscle force [33]

The net knee moments as obtained from the ID analysis revealed that the CP children had a steeper knee moment-angle curve (Fig. 2a, dashed lines), and hence overall stiffer knee extensor muscles (vasti and rectus femoris) and knee flexor muscles (hamstrings and gastrocnemius). These measured net knee moment-angle curves could almost perfectly be replicated by optimizing K and S for hamstrings and vasti (Fig. 2a, solid lines). The fit for CP was slightly better than for TD, with an RMS error between the measured and modeled knee moment of 0.10 ± 0.06 Nm for CP and 0.24 ± 0.12 Nm for TD (*p* = 0.004, Table 1).

**Table 1:** Optimization and forward dynamic simulation results

		TD (*N* = 9)	CP (*N* = 11)	Effect size	*p*-value TD vs CP
		mean ± std	mean ± std		
Muscle properties					
	S hamstrings	0.77 ± 0.15	0.57 ± 0.11	−0.20	0.008
	K hamstrings	3.59 ± 0.63	4.65 ± 1.27	1.07	0.057
	S vasti	4.21 ± 8.19	1.40 ± 1.73	−2.82	0.080
	K vasti	16.12 ± 15.28	8.73 ± 3.95	−7.40	0.322
	optimization error (Nm)	0.24 ± 0.12	0.10 ± 0.06	−0.14	0.004
FD RMS errors (°)					
	FD slow	3.58 ± 2.38	1.12 ± 0.95	−2.46	0.006
Stretch Only	FD fast - no spas	6.17 ± 3.60	19.20 ± 6.76	13.03	0.000
	FD fast - spas G1		14.59 ± 7.28		
	FD fast - spas G2		9,00 ± 5,08		
	FD fast - spas G4		9.61 ± 5.94		
	FD fast - spas G_opt_		5.95 ± 2.13		0.766^a^
Stretch + Hold	FD fast - no spas	10.55 ± 5.62	18.12 ± 5.81	7.57	0.016
	FD fast - spas G1		17.85 ± 9.25		
	FD fast - spas G2		15.27 ± 8.60		
	FD fast - spas G4		12.19 ± 4.75		
	FD fast - spas G_opt_		10.40 ± 4.59		0.941^a^

Abbreviations: *TD* typically developing, *CP* cerebral palsy, *S* passive muscle strain at optimum muscle force, *K* shape factor of passive force-length curve, *FD* forward dynamic results, *G1-4* stretch reflex gain factors of 1 to 4, *G*_*opt*_ optimal gain factor (0,1,2, or 4) per subject

^a^Spas G_opt_ results were statistically compared against TD no spas

Figure 2b shows the passive force-length curves for these optimized S and K values, averaged over all TD and CP subjects. The CP hamstrings had significantly lower S values compared with TD, with only 57 ± 11 % stretch at optimal muscle force versus 82 ± 20 % for TD (*p* = 0.004; Table 1). Shape factor K tended to be higher in CP (*p* = 0.057), indicating a more concave curve in CP than in TD. For the vasti, S and K both tended to be lower in CP, but these differences were not significant (*p* = 0.08 and *p* = 0.32 respectively). All muscles were found to be much more compliant than the default OpenSim Thelen muscle, except for the CP hamstring muscles.

### Forward dynamic results

**Fig. 3:**
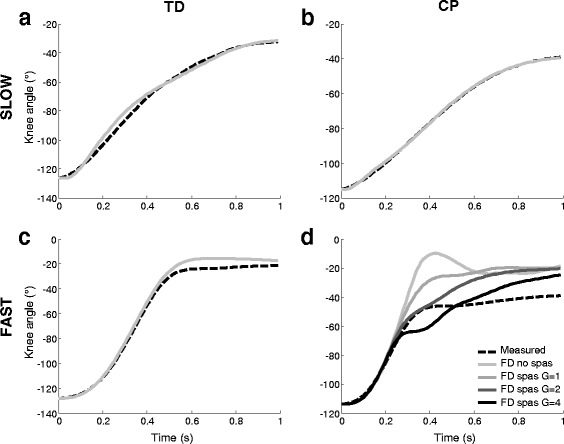
Forward-dynamic (FD) predicted (*solid lines*) versus measured (*dashed lines*) knee angles over time, for **a** typically developing (TD) slow; **b** cerebral palsy (CP) slow; **c** TD fast and **d** CP fast . Data are averaged over all subjects. For CP fast stretches, results including the stretch reflex controller with increasing gain (G) values are shown

The knee angle curve during slow passive stretch was almost perfectly replicated in the FD simulations for all CP children, with an RMS error of 1.1 ± 0.9° (Fig. 3b), thus indicating that predictive FD simulations of the clinical tests were accurate when the correct parameters were chosen. For the TD children, the fits were slightly less good, but still reasonable (RMS error of 3.6 ± 2.4°, Fig. 3a).

Even though passive muscle stiffness was not optimized for the fast stretches, the FD simulated knee angles for TD matched well with the measured knee angles, except for on average a small overshoot towards the end range of motion (Fig. 3c). The RMS error for the stretch phase only (until 0.6 s in TD) was 6.2° ± 3.6° (Table 1). For all but one CP subjects, the FD simulations without spasticity were clearly overshooting the measured knee angle (Fig. 3d). Adding spasticity reduced the overshoot and improved the fit considerably. Table 2 shows the RMS error values of predicted versus measured knee angles during fast stretches in children with CP obtained with different gain values in the stretch-reflex controller. The optimal gain, i.e. the gain with the lowest RMS error during the stretch phase (until 0.4 s in CP), is indicated per subject in bold. Most subjects had a best fit with a gain of 2 or 4, while two had a lower score with a gain of 1 and one had a best fit with a gain of zero (i.e. no spasticity). The average RMS error for predicting the knee angle with the most optimal gain value was of 5.9 ± 2.1°, which was comparable to the value for the TD children without the addition of spasticity. Towards the end range of motion, almost all subjects still had an overshoot even with the inclusion of spasticity (Fig. 3d), as also illustrated by an approximately 4° higher RMS error for the complete stretch and hold phase, compared with only the stretch phase (Table 1).

**Table 2:** Individual RMS errors (°) of predicted versus measured knee angles during the stretch phase

Subject	Gain = 0	Gain = 1	Gain = 2	Gain = 4
CP01	29.60	13.99	**5.13**	5.45
CP02	14.45	10.13	5.77	**4.85**
CP03	22.95	14.64	**7.51**	7.89
CP04	24.49	23.48	12.29	**9.06**
CP05	**8.34**	13.51	18.93	25.30
CP06	26.12	18.98	13.33	**7.41**
CP07	12.03	**6.32**	7.30	14.60
CP08	19.47	26.96	12.77	**6.41**
CP09	12.05	4.19	**2.69**	8.28
CP10	18.61	7.77	**2.49**	11.27
CP11	23.08	20.46	10.78	**5.23**

Lowest RMS-error values per subject are indicated in bold

### Muscle fiber activity, length and velocity

**Fig. 4:**
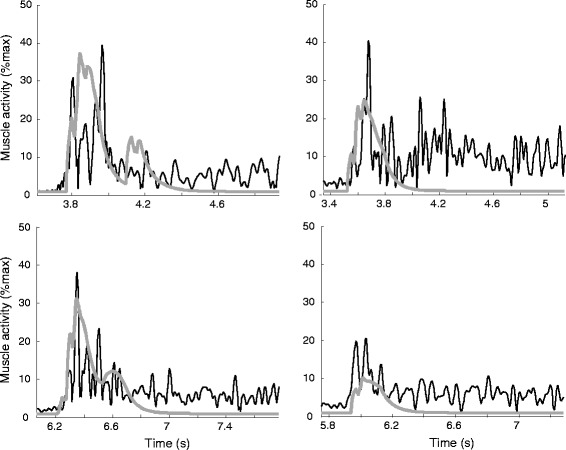
Measured EMG (*thin black lines*) and simulated (*thick grey lines*) muscle activity. Data are shown for four typical example CP children. Both EMG and simulated muscle activity are shown as a percentage of maximum, for the measured data this maximum was determined during maximum isometric contraction. For each subject, the simulation with the individually tuned optimal gain value is shown

Muscle activity came on (by definition) 30 ms after the stretch reflex controller was activated, and for all subjects the measured and simulated muscle activity was qualitatively very comparable during the main part of the stretch phase (several examples in Fig. 4). However, after the stretch phase, sustained EMG was seen in the experimental data, which was not predicted by our purely velocity-dependent spasticity model.

**Fig. 5:**
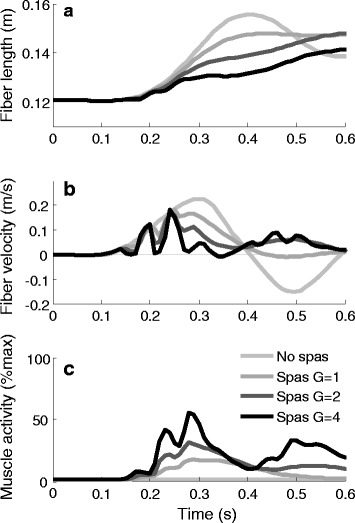
Hamstrings (**a**) fiber length, (**b**) fiber velocity and (**c**) muscle activity. Data are shown for one typical example CP subject, for simulations without spasticity and with increasing gain (G) values. Muscle activity is normalized to maximum isometric contraction

The sudden increase in muscle activation as illustrated in Fig. 4 resulted in a clear break in the muscle fiber length and velocity and thus in a lower peak fiber length and peak fiber velocity, as illustrated for a typical example CP subject in Fig. 5. With increasing gain values, more spiky patterns in length, velocity and activity were found.

## Discussion

In this study, an individually tunable, conceptual model of contracture and spasticity was developed, and tested on instrumented clinical assessment of the hamstrings in children with CP and TD children. It was found that increased resistance during slow passive stretch in both TD and CP could be simulated well by only adjusting passive muscle stiffness. Fast passive stretch could be reasonably well replicated for TD, while the addition of a purely velocity-dependent stretch reflex controller significantly improved the fast stretch simulations in CP.

Muscle stiffness parameters S and K were highly variable between subjects and substantially different in both TD children and children with CP from the generic muscle model parameters derived from Thelen [33]. Particularly for the vasti, we found a gross underestimation of the original values indicating excessive passive stiffness in the generic model. This is in line with previous studies also showing excessive passive stiffness of the quadriceps for this same model [37, 38], and the present results emphasize the magnitude of this discrepancy. In the gastrocnemius, the opposite has been reported, with the generic model indicating less passive stiffness than experimentally determined for both TD and CP [39]. These findings affirm the importance of individually tuning model parameters that aim to explain healthy and pathological pediatric muscle properties.

In line with previous findings [40], we did not find significant differences between CP and TD children for the passive stiffness properties of the vasti. This may be due to the large spread in the data and the difficulty of accurately estimating these parameters separately, especially for TD. This was likely related to the fact that the vasti muscles were not fully stretched to their end range of motion in our experimental data. For this reason, S and K were more dependent on each other for the vasti than for the hamstrings, as the stiffness curve was only in its lag phase and not shaped enough to give a good estimate for both S and K. As our focus was on the hamstring muscles, the vasti stiffness parameters were mainly used to allow for good predictive simulations. To better estimate the stiffness parameters for the quadriceps, measurements up to full knee flexion should be included.

Unlike previous modeling studies [39, 41], we opted to alter the passive properties of the hamstrings and vasti muscle tissue rather than altering its gross morphology. Specifically for the medial hamstrings, Smith et al. [16] has shown that increased collagen content in the muscles’ extra cellular matrix is a main contributor to increased muscle stiffness. Furthermore, examination of biopsies taken from the flexor carpi ulnaris of children with CP and TD children indicated significant increases in the connective tissue reinforcement of neurovascular tissues penetrating the muscle [42]. Yet, several studies have also reported that muscles from subjects with CP have reduced muscle volumes, shorter muscle bellies and shorter optimal fiber lengths compared to those of TD children [7, 16, 43]. Our finding that altered passive muscle stiffness could fully explain the experimentally measured moment angle curves during slow stretch, indicates that our contracture model may capture all these underlying causes of passive muscle stiffness, without changing the active muscle behavior.

During fast passive stretch, altering the passive muscle properties alone resulted in a reasonable simulation in TD, but not in CP. The small overshoot seen in TD (Fig. 3c) may be due to viscosity, which was not included in our model. Reflex activity is not likely to play a role in TD fast stretches, as no notable EMG activity was seen in any of the TD subjects, and hence this was not included in our simulations. In CP, a much larger overshoot was seen during fast passive stretches, and adding velocity-dependent reflex activation to the hamstrings significantly improved the fit to the level of TD. This demonstrates that the purely velocity-dependent controller could predict much of the increased resistance due to fast stretch. The large variation in gain factors among the subjects (Table 2) indicates the variability in spastic reactions among children with CP. For example, one subject with CP had hardly any overshoot in his kinematics during a fast stretch, no difference between measured and simulated end range of motion, and a best fit with measured data if no spasticity was included (Table 2, CP05). Interestingly, this was also the subject with the lowest measured RMS EMG during the fast stretch and also the lowest clinical Modified Ashworth Scale (1, where all others were 1+ or higher). Thus, spasticity played only a minor role in this subject, which was confirmed by our simulations. Current clinical assessment scores, such as the Modified Ashworth, tend to cluster muscles into broad severity categories, thereby limiting their ability to differentiate between subjects or to detect response to treatment. Conversely, the proposed model allows data from the instrumented assessment to be individually tuned, therefore providing a wider range of possible outcomes with which to compare subjects or study the effect of treatment.

Prolonged muscle activation after the fast passive stretch in CP was not predicted by our model and may, together with viscosity, help explain some discrepancies between the measured and simulated kinematics during the end range of motion in CP. Such activation may be contradictory to the classical definition of spasticity as being velocity-dependent [4], but is in line with multiple experimental studies that have reported tonic activation during passive stretch of spastic muscles [44–47], and particularly in the hamstrings [15]. A possible explanation for the continued activation may be that alterations in the membrane properties of alpha motor neurons increase their sensitivity to afferent input. This in turn triggers persistent inward currents (PIC) that lead to prolonged depolarization states called plateau potentials. Following loss of normal central regulation, PIC and plateaus can result in continuous low-level motor output [48]. Alternatively, length-dependent activity, even at slow stretch, has been found during passive muscle stretch especially in the hamstrings [15], and may help explain the prolonged activity. However, the amplitude of length-dependent activity during slow stretch was low in subjects selected for the current study, so this is likely only a minor effect.

Although these different mechanisms may all play a role, we believe that model complexity should be, in first instance, avoided. A model with many parameters may fit well with the measured data (low bias), but the accuracy with which each parameter can be estimated is prone to being compromised (high variance). Additionally, when multiple dependent parameters are included in a model, optimization algorithms tend to adjust only that parameter that has the most influence on the error, even though experimental data may show otherwise. In this study, we attempted to model the measured data with as low a number of optimizable model parameters as possible, choosing those parameters that are physiologically most likely to be altered in CP. As length, velocity, and force (or acceleration) feedback are highly interdependent, distinguishing between their effects, based on externally measured data or simulations, will be difficult. Jansen et al. [26] included both length and velocity feedback in forward-dynamic simulations of gait, and found that both feedback types resulted in pathological gait characteristics as seen post stroke. The effect of length and velocity feedback were however highly similar and therefore difficult to distinguish. Similarly, the effects of increased muscle stiffness, increased tendon stiffness, and reduced fiber and tendon length are highly related and would all result in steeper joint moment-angle curves if included in the model. We chose not to alter tendon length or stiffness, as there is limited evidence that these contribute to the overall hamstrings stiffness in CP, while Achilles tendons in CP have even been found to be more compliant [49–51]. Finally, we also had to lump the hamstrings and vasti muscle groups, as many of the individual muscle parameters were interdependent, and hence it was not possible to reliably estimate them separately. The resulting lumped hamstring is not representative of any of the individual hamstrings heads on its own, but rather of the total muscle, allowing us to better understand the overall behavior. The absolute values of the model parameters are likely influenced by the path chosen and would have been slightly different had we chosen for instance the semimembranosus or semitendinosus path. However, the differences between CP and TD are not expected to be influenced by this effect, and our conclusions are therefore independent of the muscle path chosen.

In future studies, richer experimental data sets, for instance including active force generating tests and ultrasound measurements, could provide additional information about the underlying muscle properties and behavior. This could allow for more accurate estimation of additional model parameters such as fiber and tendon lengths and active muscle properties, as well as a better distinction between the individual hamstrings heads. Simulation of additional data sets per muscle, application to other joints and muscles, and inclusion of a larger sample of subjects could also help to further validate our conceptual model for contracture and spasticity. If complemented with personalized skeletal geometry, for instance as obtained from MR images [52, 53], comprehensive patient-specific models could be generated.

As such, our individually tuned model for contracture and spasticity could be applied to simulate more clinically relevant tasks such as gait. In this way, the model may help identify to what extent passively measured (pathological) muscle properties and motor control affect dynamic tasks, and should be subject to treatment. To help speed up such applications, the stretch reflex controller as well as the underlying source code are made freely available online on the simtk website [34].

## Conclusion

This paper shows that passive muscle properties, i.e. muscle strain S and shape factor K, of hamstrings and vasti can be individually estimated based on slow stretch instrumented spasticity assessment data, and can fully explain muscle behavior during slow passive stretch. Hamstring muscle properties were found to be stiffer in CP and variable between subjects. A purely velocity-dependent spasticity model (individually tuned for its Gain) can predict muscle response during fast passive stretch in terms of predicted knee angle, muscle activity, fiber length and velocity. Only after the fast stretch phase, simulated and measured knee angles and muscle activity started to deviate, as sustained muscle activity, not dependent on velocity, was not predicted by our model.

## Abbreviations

BFS, Biceps femoris short head; CP, Cerebral palsy; DOF, degrees of freedom; EMG, Electromyography; FD, Forward dynamics; GAS, Gastrocnemius lateralis and medialis; GMFCS, Gross Motor Function Classification System; HAM, Hamstrings muscle group; ID, Inverse dynamics; K, KshapePassive, exponential shape factor for the passive force-length relationship; PIC, persistent inward currents; RF, Rectus femoris; RMS, Root mean square; S, FmaxMuscleStrain, passive muscle strain at maximum isometric muscle force; TD, Typically developing; VAS, Vasti muscle group

## Additional file

Additional file 1: Individual subject data. (XLSX 22 kb)
